# Supplementary material for: Comparative Genomics of Bryopsis hypnoides: Structural Conservation and Gene Transfer Between Chloroplast and Mitochondrial Genomes
Source: Biomolecules. 2025 Feb 13;15(2):278. doi: 10.3390/biom15020278 (PMC11852573; doi:10.3390/biom15020278)
Supplement: Supplementary file 1 [file biomolecules-15-00278-s001.zip › Figures S1-S6.pdf]

SUPPLEMENTARY FIGURES

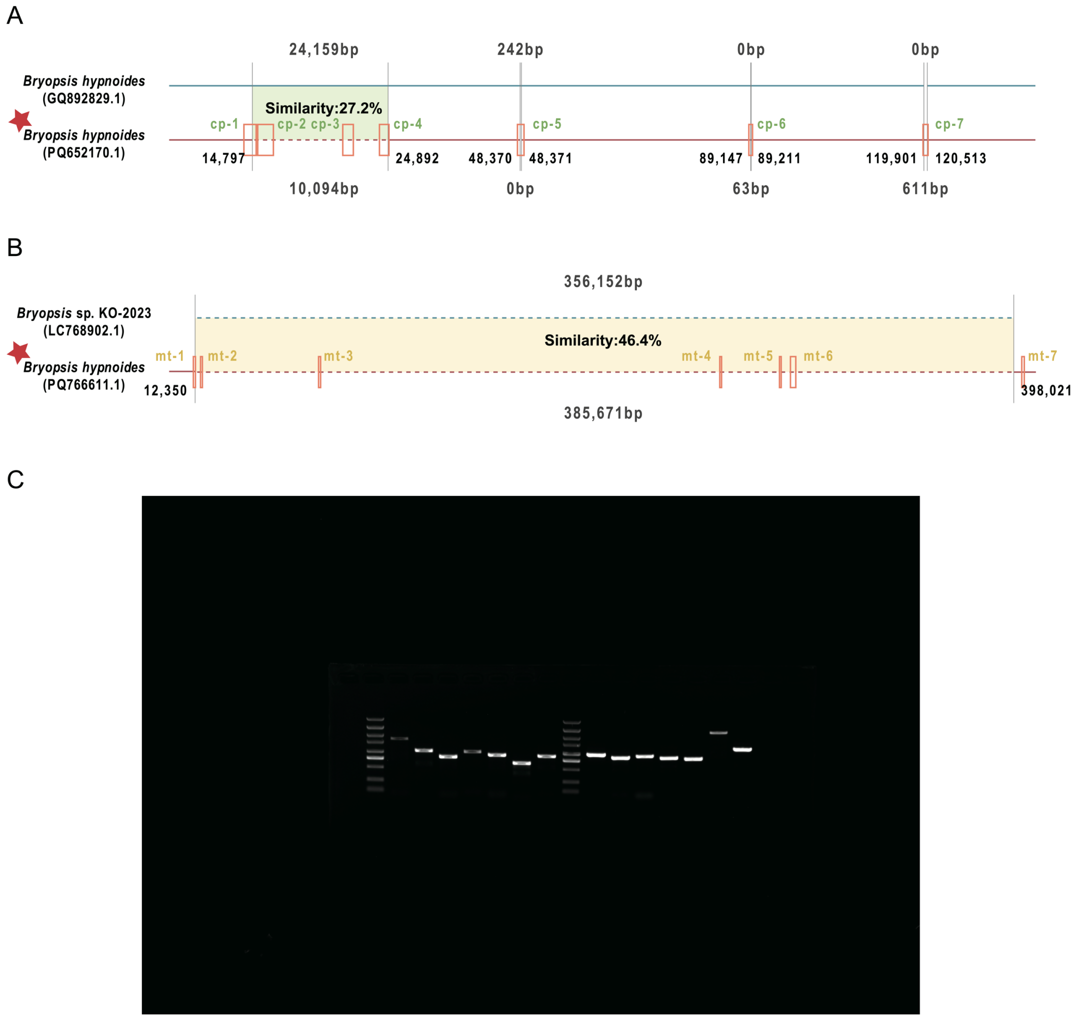

Figure S1. Primer positions and original western blot image. (A,B) Schematic representation of *B. hypnoides*' chloroplast (A) and mitochondrial (B) genomes sequence alignment with reference genomes and primer positions. The blue line represents the reference genome, the red line represents our assembled genome, and the solid and dashed lines indicate the sequence alignment result. The vertical gray line divides the genome into different regions by sequence alignment result, the length of the region is indicated by the gray number above (or under) the line, and the black number next to the line represents the position on our assembled genome. Similarity values in the light green (or yellow) areas are annotated. The orange boxes represent the designed PCR primer sites. (C) Original western blot image of PCR.

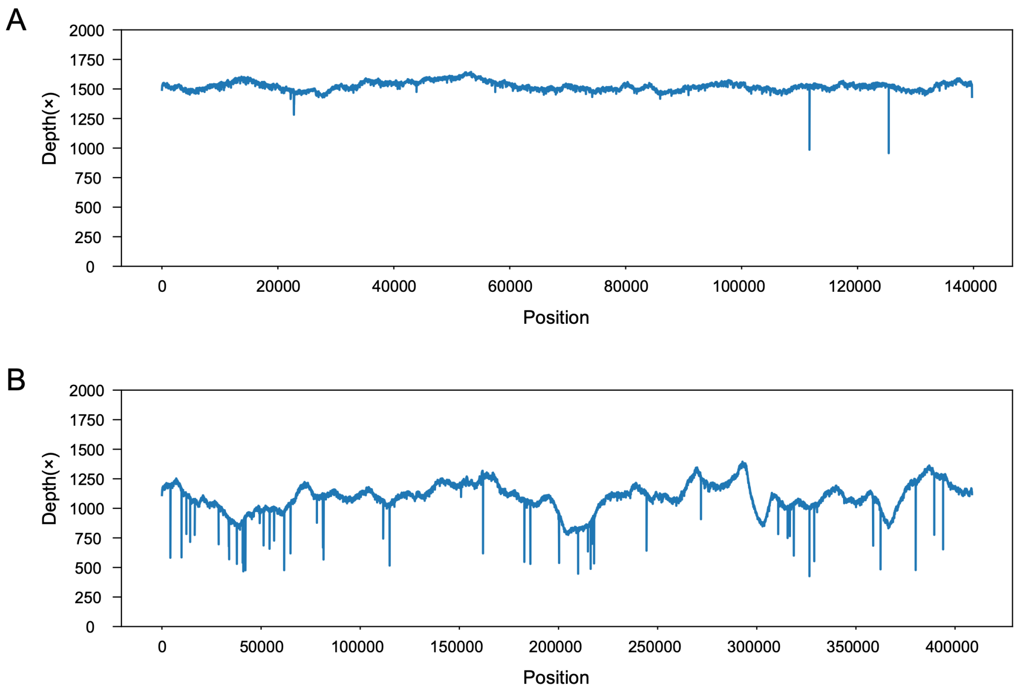

Figure S2. Overall coverage depth of the chloroplast (A) and mitochondrial (B) genomes assembly of *B. hypnoides*. The horizontal coordinate is the position of the organellar genome, and the vertical coordinate is the coverage depth.

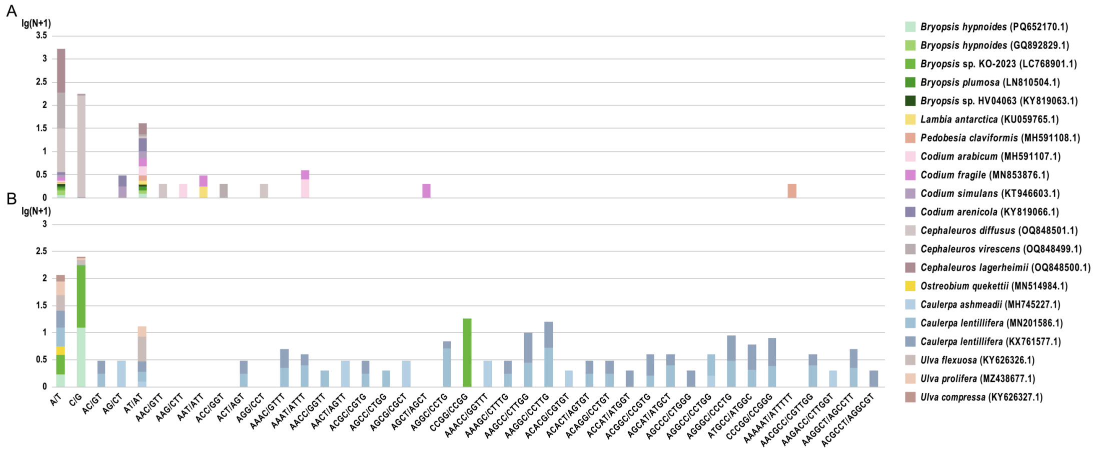

Figure S3. The different types of SSRs in the chloroplast (A) and mitochondrial (B) genomes of *B. hypnoides*. Different colors represent different species. lg(N+1) as the ordinate, N be the number of repeats of each type.

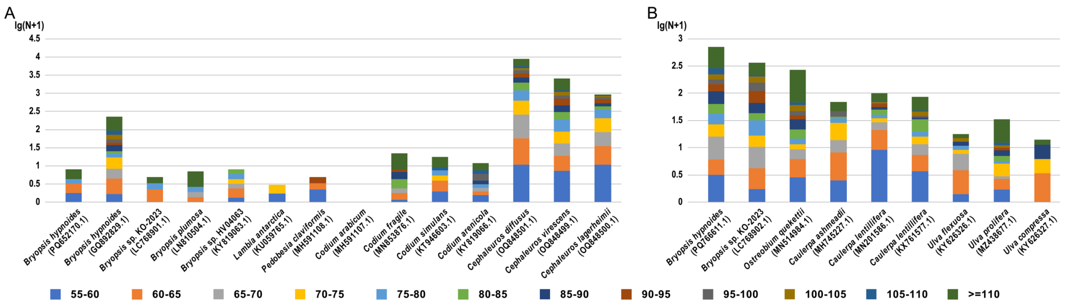

Figure S4. The different lengths of dispersed repeat sequences in the chloroplast (A) and mitochondrial (B) genomes of *B. hypnoides*. lg(N+1) as the ordinate, N be the number of repeats of each type.

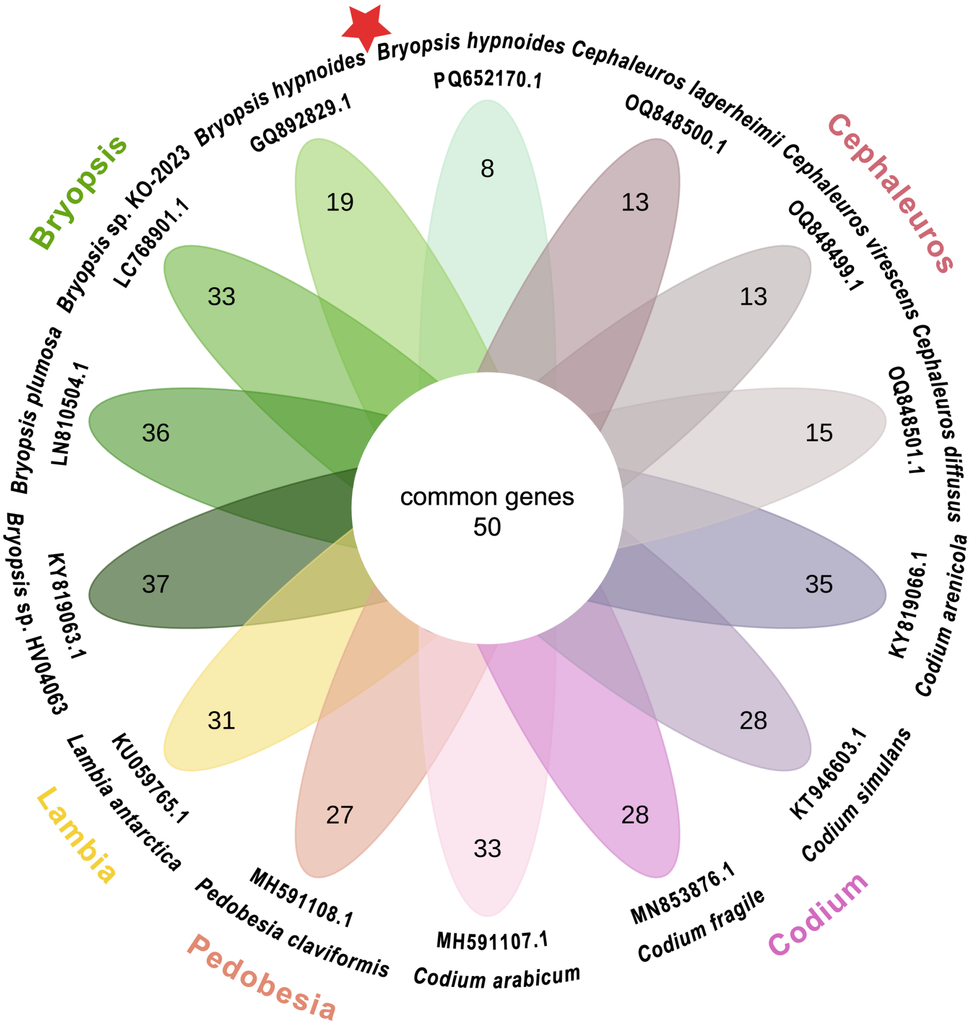

Figure S5. The distribution of the species-level common genes in chloroplast genome between different species.

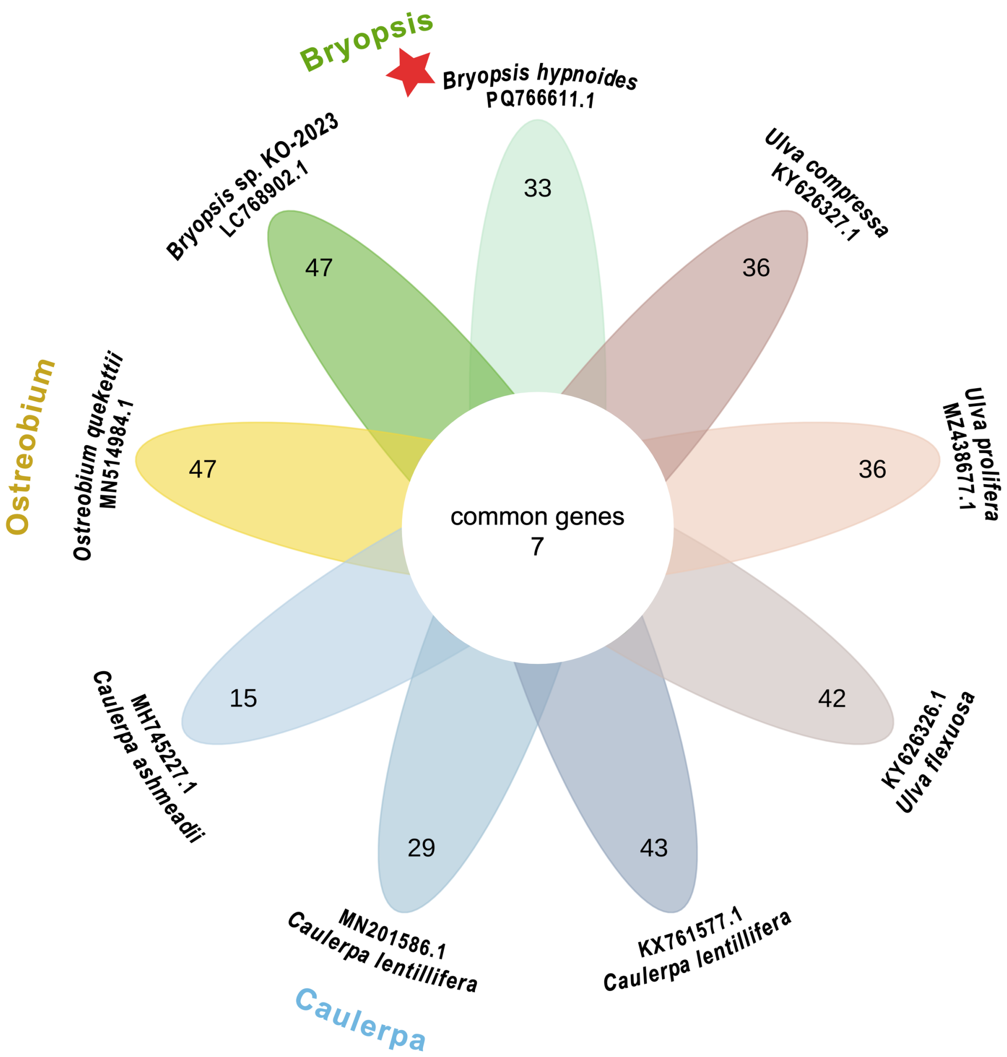

Figure S6. The distribution of the species-level common genes in mitochondrial genome between different species.

SUPPLEMENTARY TABLES

Table S1. Specific information on PCR primers.

Table S2. Information of SSRs of the chloroplast genome of *B. hypnoides.*

Table S3. Information of SSRs of the mitochondrial genome of *B. hypnoides.*

Table S4. Information on specific types of SSRs in chloroplast genome of *B. hypnoides.*

Table S5. Information on specific types of SSRs in mitochondrial genome of *B. hypnoides.*

Table S6. Information on lengths of dispersed repeat sequences in chloroplast genome of *B. hypnoides.*

Table S7. Information on lengths of dispersed repeat sequences in mitochondrial

genome of *B. hypnoides.*

Table S8. Statistics of codon usage of chloroplast genome.

Table S9. Statistics of codon usage of mitochondrial genome.

Table S10. Details of gene transfer sequence between organellar genomes.
